# Associations between countertransference reactions towards patients with borderline personality disorder and therapist experience levels and mentalization ability

**DOI:** 10.47626/2237-6089-2020-0025

**Published:** 2021-05-08

**Authors:** Poornima Bhola, Kanika Mehrotra

**Affiliations:** 1 Department of Clinical PsychologyNational Institute of Mental Health and Neuro SciencesBangaloreIndiaDepartment of Clinical Psychology, National Institute of Mental Health and Neuro Sciences, Bangalore, India.

**Keywords:** Countertransference, borderline personality disorder, mentalization, psychotherapy, psychotherapists

## Abstract

**Objective:**

This exploratory study locates countertransference as a pan-theoretical concept, comprising of thoughts, feelings, and behaviors expressed or experienced by therapists toward their patients. It aims to understand the patterns of countertransference experienced in working with borderline personality disorder. Associations between countertransference reactions and therapist-related variables of experience and mentalization ability are also examined.

**Method:**

Psychotherapists (n = 117) completed the Therapist Response Questionnaire to assess patterns of countertransference experienced with a representative patient diagnosed with borderline personality disorder. They also completed a measure of mentalization ability that examined self-related mentalization, other-related mentalization, and motivation to mentalize.

**Results:**

The profile of responses across eight countertransference dimensions is discussed, with the most strongly endorsed reactions being positive/satisfying, parental/protective, and helpless/inadequate. More experienced therapists reported less negative countertransference reactions in select dimensions. Therapists’ self-reported ability to reflect on and understand their own mental states was negatively correlated with a range of difficult countertransference experiences. There were few associations between their ability to make sense of others’ mental states, the motivation to mentalize, and the strength of their countertransference reactions.

**Conclusion:**

The implications for countertransference management as well as therapist training and development are highlighted.

## Introduction

The increasing focus on the relational aspects of the therapeutic encounter^1^ has drawn attention to countertransferential reactions that may well be inevitable in the process of therapeutic work.^2^ Countertransference, originally embedded in the psychoanalytic tradition as unconscious, conflict-based responses to the client’s transference,^3^ has undergone conceptual shifts and expansions. More recently, countertransference is seen as a pan-theoretical construct, relevant across theoretical orientations.^4,5^ The contemporary expanded view of countertransference includes therapists’ conscious and unconscious reactions to the client (sensory, affective, cognitive and behavioral).

These reactions could also be “induced” among psychotherapists as a response to the presenting concerns, personality characteristics, or interpersonal styles of clients, beyond the psychotherapists’ own internal conflicts.^6^ Theoretical literature and anecdotal accounts from clinicians suggest that working with certain client groups, e.g. borderline personality and antisocial personality,^7,8^ evokes strong and distinct patterns of countertransference reactions. Borderline personality disorder is classified as a Cluster B personality disorder, along with antisocial, histrionic, and narcissistic personality disorders; all of which are marked by frequent dramatic, emotional, or erratic behaviour.^9^ Individuals with BPD can be challenging; with frequent manifestations of emotion dysregulation, the use of primitive defenses like splitting, relational disruptions, and difficulties in maintaining boundaries in the therapeutic interaction.^10^ Intense and difficult emotional reactions; anxiety, guilt, rage, helplessness, worthlessness, rescue fantasies, and even terror, have been reported in work with patients with borderline personality disorder,^11^ who “often evoke a sense of walking on eggshells” among therapists.^10^ These emotional reactions can become therapeutically counterproductive, if they are unrecognized and unexplored.

There is limited empirical support for these anecdotal accounts about countertransference reactions in therapeutic work with borderline personality disorder. A few studies have used analogue methods that assessed therapist responses to patient vignettes of depression and borderline personality disorder.^12,13^ Both these studies documented more intense and negative feelings evoked in response to written or audio vignettes of borderline pathology. A few empirical studies examined countertransference reactions with personality disorders and their relationships with therapeutic process, outcome, or selected therapist variables. Rossberg et al.^14^ reported distinct and more negative and varied emotional responses on the Feeling Word Checklist-58 to patients with Cluster A (paranoid, schizoid, and schizotypal) and Cluster B personality disorders, when compared with Cluster C (avoidant, dependent, and obsessive-compulsive) personality disorders. Negative countertransference reactions were linked with treatment dropout and less therapeutic change. Bourke and Grenyer^15^ used the Core Conflictual Relationship Theme-Leipzig/Ulm method (CCRT-LU)^16^ to examine therapists’ narratives of their relationships with patients diagnosed with borderline personality disorder (BPD) or with major depressive disorder (MDD). In sharp contrast to MDD patients who were perceived to be ‘attending’ within sessions, there were more negative responses towards patients with BPD, who were experienced as typically ‘withdrawing’. In a comparison of therapist emotional responses to representative patients with varied personality disorders, Colli et al.^17^ found several distinct response patterns, assessed on the Therapist Response Questionnaire,^18^ evoked by different forms of personality pathology. For instance, borderline personality disorder was related to helpless/inadequate, overwhelmed/disorganized, and special/over-involved countertransference reactions reported by therapists. In a study comparing countertransference patterns among adolescents with eating disorders and those with Cluster B presentations,^19^ hostile/mistreated, helpless/inadequate, disengaged, and overwhelmed/disorganized reactions were more evident in therapy with Cluster B. These few studies suggest that specific constellations of emotional responses may be evident in therapeutic work with borderline personality presentations.

Current perspectives on countertransference suggest that the reasons for these reactions are not necessarily located only within the client, and could also stem from aspects of the therapist, or the therapy situation, e.g. termination.^4^ The therapist is increasingly seen as an active ingredient in therapy, not relegated to being a passive dispenser of therapy techniques.^20-22^ Research on countertransference has begun to focus on personal and professional characteristics of therapists, as they interact with the patient’s personality and experiences.^23^

Therapist experience could be one of the therapist characteristics that could interplay with the emotional demands and challenges of working with borderline personality disorder. There could be various reasons why lack of experience could play a role in the emergence of difficult countertransference reactions. Trainees or early career practitioners often have limited exposure to patients and the range of psychopathology, and particularly borderline presentations. They might have an inadequate understanding of their own vulnerabilities, and could experience difficulties in relation to anxiety management, case conceptualization, and other technical skills.^24^

However, there is inadequate research to confirm if relatively inexperienced clinicians consistently experience specific types and intensities of countertransference reactions to patients with BPD. In the analogue study by Brody and Farber,^12^ students and interns reported significantly higher ratings of envisaged irritation to case vignettes describing a patient with BPD, compared with licensed practitioners.

Early descriptions of how to manage countertransference manifestations have focused on the therapists’ capacity to understand internal processes of the self and other.^25,26^ Therapists’ mentalization ability; the ability to understand others’ behaviors (beliefs, desires, feelings, and memories) and the ability to reflect on one’s own states of mind^27^ could have important implications for the development, recognition, and management of countertransference.^28,29^ Individuals with borderline pathology have been described as having prominent difficulties in mentalization,^30^ particularly during affectively charged interactions both within and outside the therapy room. With this vulnerable group, the therapist’s ability to envision and mentalize about the emotional reactions and life experiences of patients, and engender the same capacity in the patient, as well as to be aware of their own mental states, might be particularly important.^30^

While some research suggests that therapists’ mentalizing capacity is associated with better therapeutic outcome,^31^ there is little empirical evidence of links with patterns and intensity of countertransference reactions.

The literature review suggests that the study of patterns of countertransference in therapeutic work with borderline personality disorder is an important, yet under-examined, area of research. Therapist awareness of countertransference responses is considered essential to the therapy process^32,33^ and can provide crucial information about the patient’s internal world, while helping therapists modulate their own emotions and behaviors. When countertransference reactions are unrecognized and unaddressed, this can increase the possibility of negative therapeutic outcomes and transgression of professional boundaries.^1^

The present study could help us to understand the nuances of how countertransference emerges in therapeutic work with borderline personality disorder and substantiate its association with select therapist attributes. The findings of this exploratory study could have implications for therapist training, supervision, and professional development. The results could also provide a preliminary research base for future examinations of a range of client and therapist attributes potentially associated with countertransference manifestations.

Three research questions guided this study. The first research question was: What are the patterns of countertransference experienced in working with borderline personality disorder? The next two research questions examine the links between therapist variables and the intensity and patterns of countertransference reactions during therapeutic work with borderline personality disorder. The second research question was: Is therapist experience associated with countertransference reactions? The third research question was: Are therapist perceptions of their ability to mentalize, in relation to both self and others, associated with countertransference reactions?

## Methods

### Participants

The participants included 117 psychotherapists, with a minimum of one year experience, who had worked with at least two clients with borderline personality disorder in the past two years. The majority of the therapists were female (77.8%) and their ages ranged from 24 to 70 years (mean = 36.4 years, standard deviation [SD] = 10.64). Most therapists were clinical psychologists (83.8%), and the remaining identified as psychiatrists (9.4%), psychiatric social workers (3.4%), counselors (1.7%), and psychiatric nurses (1.7%). About three-fourths of the participants (74.4%) were employed or in private practice, while 23.1% were in doctoral training. The mean experience as a therapist was 10.82 years (SD = 8.97; range 1-41 years) and therapists reported a current average of 15 therapy sessions per week (SD = 10.2; range 2-50 sessions). These therapists had varied levels of experience in working with borderline personality disorders in their professional careers; the mean number of patients was 30 (SD = 70.2; median = 10). The therapists were based across eight countries including India, United Kingdom, United States of America, Australia, Finland, Italy, New Zealand, and Singapore; a majority (87.2%) were from India.

The therapists responded to the study measures with reference to their most recently seen therapy patient with borderline personality disorder. These representative patients (n = 117) were predominantly female (87.2%), with a mean age of 27.4 years (SD = 8.28; range = 17-57 years). Comorbid diagnoses, including depression, substance abuse, anxiety disorder, obsessive compulsive disorder, eating disorder, and adult attention deficit disorder were documented for 87.2% of the patients. The mean duration of therapy with these patients was 1.09 years (SD = 1.45) at the time of the study, and ranged widely between 1 month and 10 years. The therapeutic work with the identified patient was typically informed by more than one theoretical orientation, used in varying degrees (reported using a 5-point scale, 0 = not at all, 5 = greatly). The most strongly endorsed approaches included dialectical behavior therapy, cognitive-behavior therapy, supportive therapy, and psychodynamic therapy.

About half (54%) of the therapists reported receiving supervision during therapy with the identified patient.

### Measures

#### Clinical data sheet

This questionnaire was constructed to obtain sociodemographic and professional information from the therapists who participated in the study and details about their most recently seen patient with a diagnosis of borderline personality disorder. Therapists provided information about their age, gender, educational qualifications, current training or employment status and professional identity (clinical psychologists, psychiatrists, psychiatric social workers, counselors, and psychiatric nurses) and the country in which they were residing and working. They also provided information about their therapeutic work; years of experience as a therapist, current number of therapy sessions per week, and total number of patients with borderline personality disorder seen in therapy. They provided details about their most recent patient with borderline personality disorder (age, gender, and comorbid diagnoses if any) and about their therapeutic work with this patient (duration of therapy and access to therapy supervision), and degree to which the therapy was guided by different approaches (rated from 0 = not at all to 5 = very greatly). The therapists rated nine therapeutic approaches; psychodynamic/transference-focused therapy, dialectical behavior therapy, cognitive-behavior therapy, mentalization-based therapy, schema-focused therapy, systems training for emotional predictability and problem solving, supportive therapy, humanistic/existential therapy and interpersonal therapy, and an ‘other’ category to be specified.

## Therapist Response Questionnaire (TRQ)^18^

This 79-item self-report measure assesses a wide spectrum of thoughts, feelings, and behaviors expressed or experienced by therapists toward their patients. These range from relatively specific feelings (e.g., “I feel bored in sessions with him/her”) to complex constructs, such as projective identification (e.g., “More than with most patients, I feel like I’ve been pulled into things that I didn’t realize until after the session was over”). Each item is individually rated using a five-point Likert scale (1 = not true at all, 3 = somehow true, and 5 = fairly true). The items are written in a way that they can be responded to by therapists of any theoretical orientation.^34^

The factor structure of the TRQ comprises eight countertransference dimensions^34^:

- overwhelmed/disorganized (9 items), indicates a desire to avoid or flee the patient and strong negative feelings, including dread, repulsion, and resentment;
- helpless/inadequate (9 items), describes feelings of inadequacy, incompetence, hopelessness, and anxiety;
- positive (8 items), indicates the experience of a positive working alliance and close connection with the patient;
- special/over-involved (5 items), describes a sense of the patient as special, relative to other patients, and includes ‘soft signs’ of problems in maintaining boundaries, including self-disclosure, ending sessions on time, and feeling guilty about, responsible for, or overly concerned about the patient;
- sexualized (5 items), describes sexual feelings toward the patient or experiences of sexual tension;
- disengaged (4 items), describes feeling distracted, withdrawn, annoyed, or bored in sessions;
- parental/protective (6 items), is marked by a wish to protect and nurture the patient in a parental way, above and beyond normal positive feelings toward the patient;
- criticized/mistreated (18 items), describes feelings of being unappreciated, dismissed, or devalued by the patient.

The scales’ scores are obtained by calculating the average score of the items that make up each countertransference factor.

The TRQ has good psychometric properties; excellent internal consistency of the eight factors and good convergent validity, Exploratory and confirmatory factor analyses of data from an Italian version, revealed 9 distinct countertransference factors that were similar to 8 dimensions identified in the original version of the measure.^35^ In the present study, Cronbach’s alpha for internal consistency ranged from acceptable (0.6 < α < 0.7) to good (0.7 < α < 0.9) and excellent (α > 0.9).^36^ The estimates were: overwhelmed/disorganized (α > 0.83), helpless/inadequate (α > 0.91), positive/satisfying (α > 0.81), special/over-involved (α > 0.70), sexualized (α > 0.60), disengaged (α > 0.69), parental/protective (α > 0.80) and criticized/mistreated (α > 0.92).

In the present study, each participant was asked to select their most recently-seen patient with borderline personality disorder and respond to the TRQ.

### The Mentalization Scale (MentS)^37^

This 28 item measure assesses mentalizing capacity, and has three sub-scales; self-related mentalization (MentS-S), other-related mentalization (MentS-O), and motivation to mentalize (MentS-M). Each item is individually rated using a five-point Likert scale (1 = completely incorrect to 5 = completely correct). The psychometric properties of the MentS, have been examined in studies with employed adults and university students from the general population and with a clinical sample of persons with borderline personality disorder (BPD). The internal consistency of the scale was good in the community and acceptable in the clinical sample (α = 0.84 and 0.75, respectively). The three subscales were identified through principal components analysis and had acceptable reliabilities (α = 0.74-0.79), except for MentS-M in the clinical sample (α = 0.60). MentS scores further exhibited a coherent pattern of correlations; relating positively to empathy, trait and ability emotional intelligence, openness, extraversion, and conscientiousness, and negatively to attachment avoidance and anxiety, and neuroticism. In the present study, internal consistency estimates were good (0.7 < α < 0.9)^36^ for all three subscales; MentS-S (α = 0.81), MentS-O (α = 0.72), and MentS-M (α = 0.73).

## Procedure

This study was approved by the ethics committee at the National Institute of Mental Health and Neuro Sciences. Mental health practitioners across India were contacted through professional association mailing lists or alumni association contacts. Participation from international participants was sought though the mailing list of the Society for Psychotherapy Research. A secure link to access an online version of the survey was provided in the email. The options for SSL encryption and masking of IP address were enabled for the online survey, hosted on the SurveyMonkey platform. There was a “no response” option for every question, and the participants had the option to withdraw from the survey before finally submitting their responses. Participants who indicated a preference for a paper version (24.8%), were provided with envelopes for separate return of the consent form and questionnaires. There was no compensation offered in exchange for participation and no identifying data were collected about the representative patients.

## Data analysis

Descriptive statistics (mean, SD, and frequencies) and inferential statistics were used to analyze data. Bivariate correlations were computed to examine associations between countertransference dimensions (TRQ) and therapist experience levels and mentalization (MentS). T tests were used to assess differences in countertransference dimensions between therapists who received supervision and those who did not.

## Results

The pattern and intensity of therapist countertransferential responses in their work with a representative patient with borderline personality disorder are reported in Table 1.

**Table 1:** Means and standard deviations of the TRQ dimensions

TRQ dimensions	M	SD
Positive/satisfying	2.88	0.77
Parental protective	2.54	0.74
Helpless/inadequate	2.43	0.77
Overwhelmed/disorganized	2.05	0.66
Criticized/mistreated	2.00	0.60
Disengaged	1.86	0.60
Special/over-involved	1.85	0.63
Sexualized	1.14	0.26

M = mean; SD = standard deviation; TRQ = Therapist Response Questionnaire.

The results (Table 1) indicated that therapists experienced a range of cognitive, affective, and behavioral responses to their patients. The most strongly endorsed reactions reflected a positive connection and engagement with the patient (positive/satisfying), and nurturant parental feelings that went beyond what is usual or typical (parental protective). Therapists also reported feelings of helplessness, inadequacy, and anxiety in the therapeutic interactions with their patient (helpless/inadequate), and strong negative feelings accompanied by the desire to avoid or end the interaction (overwhelmed/disorganized). Other countertransference reactions (feeling criticized/mistreated, disengaged, or sexualized feelings in the interaction) were less strongly endorsed by therapists in this sample.

The associations between therapist variables, experience level and mentalization ability, and countertransference reactions in eight dimensions of the TRQ are reported in Table 2.

**Table 2:** Bivariate correlations between TRQ dimensions, therapist experience levels and mentalizing capacity (MentS)

TRQ Dimensions	Experience (years)	Experience (BPD)	MentS-self	MentS-other	MentS-motivation
Criticized/mistreated	-0.06	-0.16	-0.38*	-0.17	0.03
Helpless/inadequate	-0.20^†^	-0.22^†^	-0.34*	-0.08	0.18
Positive/satisfying	-0.02	0.09	-0.01	-0.03	0.14
Parental/protective	-0.09	-0.08	-0.20^†^	-0.17	0.19^†^
Overwhelmed/disorganized	-0.18^†^	-0.19^†^	-0.31*	-0.07	0.15
Special/over-involved	-0.25*	-0.22^†^	-0.27*	-0.01	0.12
Sexualized	0.14	-0.05	-0.12	-0.39*	-0.27*
Disengaged	-0.05	-0.23^†^	-0.24^†^	-0.14	-0.04

BPD = borderline personality disorder; MentS = The Mentalization Questionnaire; TRQ = Therapist Response Questionnaire.

* p < 0.01; ^†^ p < 0.05.

The results (Table 2) indicated that more experienced therapists reported lower levels of helplessness and inadequacy during sessions (r = -0.20, p = 0.034) and were less likely to report a sense of being overwhelmed and experiencing negative and avoidant feelings towards their patient (r = -0.18, p = 0.048). Additionally, therapists with more years of experience were less likely to give their patient special status or be over-concerned about them and over-involved with them (r = -0.25, p = 0.006).

Therapists who had seen more clients with BPD over the course of their careers also reported less negative countertransference in similar domains and less disconnection or feelings of being distracted, withdrawn, annoyed, or bored in sessions (r = -0.23, p = 0.015). The magnitudes of correlations were interpreted based on guidelines recommended by Gignac and Szodorai^38^ as small (r = 0.10-1.9), medium (r = 2.0-2.9), or large (r ≥ 0.30). Most associations between therapist experience levels and countertransference reactions ranged between small and medium effects.

In an additional analysis (Table 3), countertransference experiences were compared between two groups: therapists who had received supervision during their therapeutic work with their representative BPD patients (53%) and those who had not (47%). The results indicated that being under supervision was associated with higher levels of difficult or negative countertransference reactions; including feelings of being mistreated, helpless/inadequate, overwhelmed, and disengaged. Therapists who had received supervision also reported significantly higher parental or protective feelings about their patient. These findings are better understood in the context of years of experience; therapists who received supervision had significantly lower levels of experience (t = -3.09; p < 0.05).

**Table 3:** Comparison of TRQ dimensions between therapists who received supervision and those who did not

TRQ dimensions	Supervision n = 63		No supervision n = 53		t value

	M	SD	M	SD	
Criticized/mistreated	2.20	0.62	1.87	0.55	2.32*
Helpless/inadequate	2.59	0.75	2.26	0.75	2.37*
Positive/satisfying	2.95	0.63	2.80	0.57	1.30
Parental/protective	2.71	0.75	2.34	0.68	2.81^†^
Overwhelmed/disorganized	2.20	0.65	1.89	0.65	2.59*
Special/over-involved	1.95	0.62	1.75	0.62	1.71
Sexualized	1.11	0.25	1.16	0.27	-0.98
Disengaged	1.96	0.60	1.74	0.58	2.03*

M = mean; SD = standard deviation; TRQ = Therapist Response Questionnaire.

* p < 0.05; ^†^ p < 0.01.

Table 2 indicates that therapists’ understanding of their own mental states was negatively correlated with a range of difficult countertransference experiences; feelings of being helpless and inadequate (r = -0.34, p = 0.000), feeling unappreciated, dismissed or devalued by the patient (r = -0.38, p = 0.000), a sense of being disengaged during sessions (r = -0.24, p = 0.011), being overwhelmed by negative feelings and wishing to avoid or flee the interaction (r = -0.31, p = 0.001). Higher levels of therapists’ ability to mentalize their own actions, thoughts, and feelings were associated with lower likelihood of an over-concerned over-involved stance (r = -0.27, p = 0.004) and parental feelings (r = -0.20, p = 0.028) towards their clients with borderline presentations. The results also indicated that sexualized countertransference reactions are more likely to be present with their borderline client when therapists are less motivated to mentalize (r = -0.39, p = 0.000) or experience more difficulties in mentalization of “other’s” actions, thoughts and feeling (r = -0.27, p = 0.004). This involves a sense of sexual tension or attraction in the therapist-client dyad, or a verbal/behavioral expression of this feeling by the therapist. The significant correlation between therapists’ motivation to mentalize and parental protective feelings toward the patient (r = 0.19, p = 0.045) indicated that lower interest in understanding the reasons for behaviors, intentions and feelings, was associated with less nurturant and protective responses.

The effect sizes of the correlations between therapist reports of mentalization were between small (r = 0.10-1.9) to large (r ≥ 0.30), per Gignac and Szodorai’s^38^ benchmarks.

## Discussion

The findings illustrate the range of emotional responses that can be evoked during therapeutic work with borderline personality disorder. While the process of working with this vulnerable group can be experienced as rewarding and meaningful, it can also make therapists feel uncertain, inadequate, and confused.

Contrary to reports that intense negative feelings are dominant in therapeutic work with borderline patients,^7^ the present study found that positive, satisfying reactions were most commonly endorsed by the participant therapists. This dimension captures a sense of positivity and engagement in the therapeutic work, but also encompasses potentially difficult responses such as liking or regarding the patient as a “favorite” and feeling gratified in this interaction. Parental feelings of warmth, nurturance, anger at other people in the patient’s life, and a desire to compensate for their experiences of deprivation were also among the strongest reactions from therapists in this study. Despite the myriad challenges in working with borderline personality disorder, clinicians tend to experience a sense of therapeutic optimism and a view of the patient as “special”.^10^ However, emotional responses that are “too positive”,^5^ or behaviors that reflect therapists’ needs to protect and “rescue” their patients,^13^ have also been recognized as problematic. Positively-valenced feelings and behaviors can be associated with the risk of therapist over-involvement with the client and a loss of objectivity. Breivik et al.^39^ speculated that therapists may report more positive feelings either due to defensive processes or lack of awareness of their negative feelings towards patients with personality disorders.

Similar to the current study, previous research also described the triad of positive/satisfying, parental/protective, and helpless/inadequate as the strongest responses while working with patients with personality disorders.^40^ The experience of both negative and positive countertransference responses is not uncommon; a combination of helpless/inadequate, overwhelmed/disorganized, and special/over-involved patterns emerged in another study with borderline pathology.^17^ Perhaps such mixed responses from therapists reflect the split and shifting self-other representations among persons with borderline personality disorders.^41,42^ Betan, Heim, Zittel Conklin, and Westen^34^ suggested that a wider range of countertransference reactions may be experienced and expressed in therapeutic work with BPD, in comparison to other forms of personality pathology. Although previous research indicated that Cluster B/borderline personality pathologies were associated with distinct countertransference patterns,^17,19^ the lack of comparison groups in the present study precludes an understanding of whether these emergent countertransference reactions are characteristic of therapy with BPD.

Countertransference is increasingly being seen as a two-person phenomenon,^5^ and our study findings demonstrate how therapists’ experience levels and mentalization abilities enter the therapeutic arena. Our findings suggest that accumulating therapeutic experience over the career trajectory and seeing more clients with borderline personality disorder makes practitioners less susceptible to difficult countertransference reactions. This is in line with previous research^12,13,23^ which has also indicated that more seasoned therapists had less intense and fewer negative emotional reactions to borderline patients. However, the findings from these three studies were limited by the use of written or audiotaped case vignettes to evoke responses from participant therapists. The links between therapist experience levels and the occurrence of difficult countertransference reactions may not be restricted to BPD and similar findings have been reported with narcissistic personality disorder,^43^ another Cluster B disorder.

Therapeutic work is a meaning making process, one in which both the patient and the therapist need to represent their inner worlds in mental state terms.^44^ In this process, the therapist needs to look both inward and outward, at their own experiences and at what the patient might be doing or feeling.^5^ The study findings indicate that negative countertransference manifestations were less prominent when therapists’ reported better understanding of their own mental states. Interestingly, there were few associations between countertransference reactions and therapists’ capacity to mentalize their patients’ actions and experiences. Barreto and Mantos^44^ also indicated that therapists’ differentiated and nuanced awareness of their “own” feelings, needs, beliefs, reasons, and processes is critical in “mentalizing the countertransference”, particularly in working with BPD, where reflective processes are more likely to be challenged.^45^

Training, supervision, and continuing professional development initiatives can help trainees and practitioners monitor and track their thoughts, feelings, and behaviors, while continuing to be open and responsive in the therapeutic interaction. Discussions and sharing of relevant research evidence can help normalize the experience of difficult negative emotions, worries about competence and skills, and possible disconnection from the therapeutic process. The study findings also point to the risks of heightened responsibility, over-involvement, and potential boundary transgressions among less experienced therapists, and this would need special attention. Novice and early career therapists, who seem more vulnerable to difficult emotional reactions during therapeutic work with this challenging group, would benefit from additional support and more intensive supervision. In fact, Liebman and Burnette^23^ recommend early exposure to therapeutic work with borderline personality disorder to build therapists’ competence at a time when supervision is more consistently available. Recognition and management of countertransference can help understand psychopathology, anticipate and address difficulties and ruptures in the therapeutic alliance, track the therapy process, and address possible burnout from continued work with challenging patients.^46,47^ Awareness of repetitive countertransference reactions can point to issues that need to be addressed in supervision, personal work, or through additional training.

The associations between therapist mentalization and countertransference in the present study, the concerns about sexualized reactions and boundary transgressions with borderline personality disorder,^11^ and the emergent links between therapist reflective functioning and therapy outcome^48,49^ all point to the need to focus on therapist mentalization capacity. Although therapeutic approaches for borderline conditions, such as mentalization-based therapy^30^ and transference-focused therapy,^50^ emphasize the human encounter, countertransference, and the inner life of the therapist, the discussions on therapist mentalization capacity need more attention.^45^

This study has limitations arising from a possible sample selection bias; therapists who chose to respond to this survey may not form a representative group. The use of self-report to assess complex processes such as countertransference and mentalization has potential shortcomings. There are possible influences of memory biases and social desirability effects, apart from the difficulties in recognition of difficult countertransference experiences. Typically, therapeutic interventions for borderline personality are both intensive and long-term, and changes in the types, pattern and intensity of countertransference reactions could be expected over the course of this interaction. However, in the present study, therapists were assessed at varied stages of their work with a representative patient, with wide variations in the duration of therapy.

Our findings highlight a number of avenues that could be explored in future research. Integration of a range of variables related to therapist (e.g. gender, theoretical orientation), client (e.g. symptom severity, comorbidity, attachment style), and therapy (e.g. duration, modality, phase of therapy, other process and outcome variables) is recommended in future studies on countertransference patterns. Qualitative research involving an examination of session transcripts at different stages of the therapy process could provide additional perspectives on the intersubjective countertransference and mentalizing processes that emerge in the dyad.^51^ Studies that involve in-depth interviews with therapists could tap their awareness, experience, and strategies to manage positive and negative countertransference experiences in working with borderline personality disorder. There have been encouraging results from brief training initiatives aimed at increasing awareness, commitment to monitoring, and discussing countertransference among trainee therapists,^52,53^ and at improving therapist mentalization.^54,55^ Future research can contribute to effective training and development initiatives and assess if these are associated with therapy outcomes.

This paper was previously presented as a brief paper titled “Patterns of countertransference in psychotherapy with persons with borderline personality disorder,” at the Society for Psychotherapy Research 5th Joint European & UK Chapters conference, held in September 19-21 2019, in Krakow, Poland.
